# High level expression of the anti-retroviral protein APOBEC3G is induced by influenza A virus but does not confer antiviral activity

**DOI:** 10.1186/1742-4690-6-38

**Published:** 2009-04-16

**Authors:** Eva-K Pauli, Mirco Schmolke, Henning Hofmann, Christina Ehrhardt, Egbert Flory, Carsten Münk, Stephan Ludwig

**Affiliations:** 1 Institute of Molecular Virology (IMV), Centre of Molecular Biology of Inflammation (ZMBE), Westfaelische-Wilhelms-University Muenster, Münster, Germany; 2 Clinic for Gastroenterology, Hepatology and Infectiology, Heinrich-Heine-University, Duesseldorf, Germany; 3 Paul-Ehrlich-Institute (PEI), Langen, Germany

## Abstract

Human APOBEC3G is an antiretroviral protein that was described to act via deamination of retroviral cDNA. However, it was suggested that APOBEC proteins might act with antiviral activity by yet other mechanisms and may also possess RNA deamination activity. As a consequence there is an ongoing debate whether APOBEC proteins might also act with antiviral activity on other RNA viruses. Influenza A viruses are single-stranded RNA viruses, capable of inducing a variety of antiviral gene products. In searching for novel antiviral genes against these pathogens, we detected a strong induction of APOBEC3G but not APOBEC3F gene transcription in infected cells. This upregulation appeared to be induced by the accumulation of viral RNA species within the infected cell and occurred in an NF-κB dependent, but MAP kinase independent manner. It further turned out that APOBEC expression is part of a general IFNβ response to infection. However, although strongly induced, APOBEC3G does not negatively affect influenza A virus propagation.

## Findings

In patients infected with HIV-1, the expression of human apolipoprotein (apo) B mRNA editing enzyme catalytic polypeptide 1-like protein 3G (APOBEC3G) was observed to be elevated [1], although this was not confirmed in cell culture experiments [2,3]. Members of the APOBEC3 family are known to act with anti-retroviral activity against HIV [4,5], but they also inhibit replication of hepatitis B virus (HBV) [6], and adeno-associated virus type 2 [7]. The anti-retroviral activity of human APOBEC3 proteins is probably conferred by cytidine deamination of the newly synthesized first viral cDNA strand. This mechanism is counteracted by the HIV-1 protein virion infectivity factor (Vif) [8-12]. However, human APOBEC3 proteins may not only have anti-retroviral or anti-HBV activity. Two findings have triggered a broader interest in these proteins with regard to a potential antiviral action against RNA viruses. First, besides its DNA deamination activity, human APOBEC3 proteins were reported to also possess RNA deamination activity [13]. Second, DNA deamination activity may not be the only antiviral action of these proteins [13-16] suggesting that APOBEC3s might possess functions that render them effective against other viruses, which do not have any DNA-intermediates during replication such as influenza A virus.

**Figure 1:**
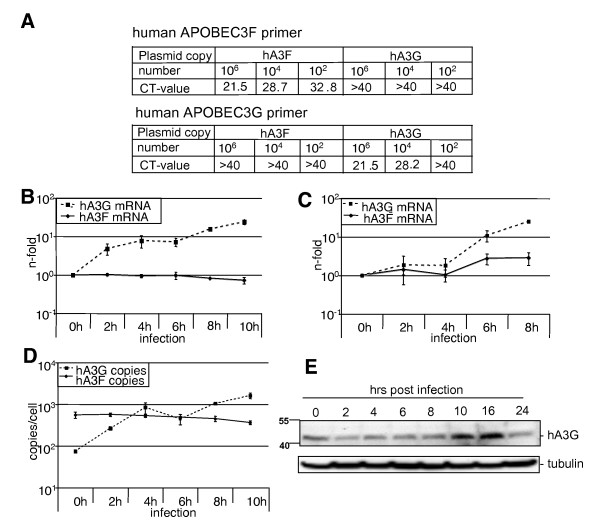
**Virus-induced human APOBEC3G gene transcription**. (A) Determination of the binding specificity of human APOBEC3F and human APOBEC3G primers in quantitative real time PCR (qRT-PCR). Serial dilutions of the C-terminally HA-tagged plasmids pcDNA_huAPOBEC3F (hA3F) (described by H. Muckenfuss and colleagues) or pcDNA_huAPOBEC3G (generously provided by Nathaniel R. Landau) were analysed using either human APOBEC3F or APOBEC3G specific primer pairs in qRT-PCR. The copy number of each plasmid and the corresponding CT-values (cycle number of the first detectable signal) are given. Low CT-values indicate high amounts of the DNA-sequence of interest. CT-values above 30 are commonly considered as non-specific signals. The qRT-PRC-program is limited to 40 cycles; due to that fact that CT-values >40 indicate undetectable amounts of DNA. (B-E) The influenza virus strain A/Puerto Rico/8/34 H1N1 (PR8) was diluted in PBS containing 0.6% sterile BSA and 1% penicillin/streptomycin and used to infect A549 cells (B and D-E) or HUVEC (C) (MOI = 5) for the time points indicated. At two-hourly intervals post infection, RNA was isolated using the RNeasy mini-kit (Qiagen), and 3 μg of total RNA were transcribed into cDNA using 0.5 μg oligo dT-primer (16 mer) and 200 U Revert Aid H^- ^(Fermentas) according to the manufacture's protocol. mRNA levels of IFNβ, human APOBEC3F and human APOBEC3G were assessed by qRT-PCR using primer pairs for human APOBEC3F and 3G; or for human IFNβ as follows: IFNβ_fwd 5'-GGC CAT GAC CAA CAA GTG TCT CCT CC-3' and IFNβ_rev 5'-GCG CTC AGT TTC GGA GGT AAC CTG T-3'. Induced transcription of mRNA was calculated as n-fold using GAPDH as reference gene. (D) Determination of the copy numbers per cell of human APOBEC3F or human APOBEC3G. (E) Infected A549 cells were lysed at the time points indicated. Endogenous expression of human APOBEC3G was determined with the hA3G specific antibody ApoC17 (NIH AIDS Research and Reference Reagent Program) in Western blots. An anti-tubulin (B5-1-2, Sigma) blot served as a loading control.

In global gene expression profiling studies of influenza A virus-infected cells, we observed strongly elevated transcription of human APOBEC3G. This finding was verified by quantitative Real-time PCR (qRT-PCR) [17] with specific primers against APOBEC3G and APOBEC3F (Figure 1A) [18-20] in lung epithelial cells (A549) (Figure 1B) and in primary human endothelial cells (HUVEC) (Figure 1C) infected with the human influenza virus A/Puerto Rico/8/34 H1N1 (PR8). The upregulation of APOBEC3G was also confirmed on protein level as determined in Western blot analysis (Figure 1E). Protein expression steadily increased with time up to 16 hours post infection but dropped again at 24 hours p.i (Figure 1E) most likely due to host cell protein shut-off induced by the virus. Interestingly, such an upregulation of human APOBEC3G transcription was not reported for cells infected with HIV-1 [2,3], although higher expression levels of human APOBEC3G in HIV-1 infected patients is described in the literature [1]. Upregulation of APOBEC3G was also confirmed in cells infected with the human H5N1 influenza virus isolate A/Thailand/(KAN-1)/2004 (H5N1) (data not shown), suggesting that transcriptional induction of APOBEC3G is a general phenomenon in influenza A virus infected cells. Interestingly, the paralogue human APOBEC3F was not found to be upregulated in A549 cells and was only marginally induced in HUVEC (Figure 1B and 1C). This is noteworthy, since human APOBEC3F and human APOBEC3G share more than 90% promoter sequence similarity and appear to be transcriptionally co-regulated [4,5]. However, co-regulated induction of expression was not observed in our experiments. Instead we found that the mRNA copy number of APOBEC3F remains at a constant high level in uninfected and infected A549 cells, while the copy numbers of APOBEC3G are at a low level in uninfected cells and rise upon viral infection (Figure 1D), suggesting distinct transcriptional regulation despite high promoter sequence similarity.

**Figure 2:**
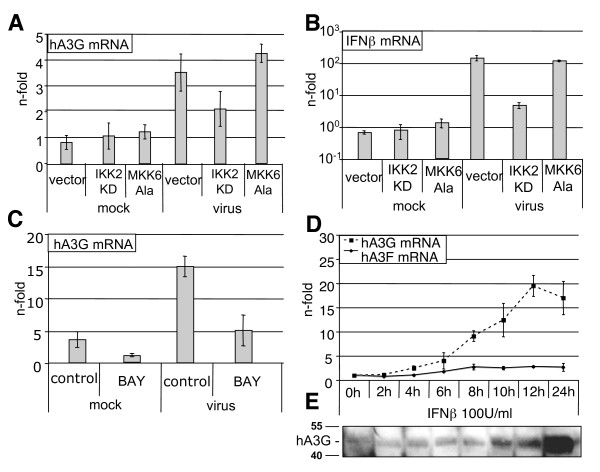
**IFNβ-induced transcription of human APOBEC3G**. (A and B) A549 cells stably overexpressing the dominant negative mutants IKK2KD or MKK6Ala. These mutant kinases were cloned in the retroviral pEGZ-vector. In this vector GFP is expressed by an internal ribosomal entry site (IRES) from the same mRNA as the IKK2KD and MKK6Ala transgenes, allowing transgene expression to be monitored by FACS-analysis (data not shown). MKK6Ala and IKK2KD overexpressing cells were infected for 10 hours with the influenza A virus strain PR8 (MOI = 5). Following infection, RNA was isolated with the RNeasy mini kit (Qiagen), reverse transcribed as described above, and cDNA was subjected to qRT-PCR. (C) A549 cells were pre-treated for 30 minutes with the NF-κB specific inhibitor BAY 11–7085 (40 μM) before infection with PR8 (MOI = 5) for 10 hours. RNA was subjected to qRT-PCR. The mRNA levels of human APOBEC3G (A and C) or IFNβ (B) were assessed by qRT-PCR. (D) A549 cells were stimulated with IFNβ (100 U/ml) (Invitrogen) for the time points indicated. The mRNA levels of human APOBEC3F and human APOBEC3G were determined by means of qRT-PCR. Induction of gene transcription was calculated as n-fold of untreated cells, which was arbitrarily set as 1, as described by Livak and Schmittgen. (E) A549 cells were stimulated as in (D), and cell lysates were subjected to Western blots using the hA3G specific antibody ApoC17 (NIH)

Given the particular strong induction of human APOBEC3G in influenza A virus-infected cells, we addressed the question which virus-induced intracellular signalling pathways are required for human APOBEC3G mRNA transcription. Influenza virus infection induces a variety of signalling pathways such as the Raf/MEK/ERK kinase cascade, the p38 signalling pathway and the IKK/NFκB pathway [21,22]. PMA, an effective inducer of the classical Raf/MEK/ERK cascade, has been reported to induce human APOBEC3G gene expression in H9 cells via PKC [23]. However, in the cell types used in our study, PMA (100–200 ng/ml) was only a weak inducer of APOBEC3G expression, and inhibition of the Raf/MEK/ERK cascade by the MEK inhibitor U0126 (2–10 μg/ml) did not result in reduced human APOBEC3G mRNA levels in virus-infected A549 cells (data not shown). Activation of the p38 signalling cascade by virus infection involves the phosphorylation of p38 by the MAP kinase kinase, MKK6. To block the pathway at this level of the cascade we overexpressed a dominant negative mutant of MKK6 (MKK6Ala) that was previously shown to efficiently suppress the activation of p38 [24]. Successful transduction of the retroviral vector pEGZ-MKK6Ala was monitored by FACS-analysis of GFP (data not shown) that is expressed from a second reading frame of the mRNA of the transgene [24]. Inhibition of the p38 phosphorylation by either stable overexpression of the dominant-negative form of MKK6 (Figure 2A) or application of the p38 inhibitor SB203580 (20 μM) (data not shown) did not affect the induced transcription of APOBEC3G. These findings argue against a prominent role of either, ERK or p38 MAPK cascade in viral APOBEC3G induction.

The IKK2/NF-κB module is another influenza virus-activated signalling cascade that is known to regulate a variety of genes. This includes IFNβ transcription, which is controlled by an enhanceosome, composed of the transcription factors IRF3/7, NF-κB, and AP-1 [22]. To assess the involvement of IKK2 and NF-κB in virus-induced APOBEC3G expression, we used A549 cells that were retrovirally transduced with the vector pEGZ-IKK2KD. This transduction allows for the stable expression of the dominant negative mutant of IκB kinase 2 (IKK2), an approach that has been successfully used previously to efficiently blunt NF-κB activity [25,26]. Upon infection of these mutant-expressing cells, APOBEC3G mRNA levels were reduced compared to control cells (Figure 2A) to a similar extent that was observed for the IFNβ gene (Figure 2B). The same pattern of APOBEC3G expression was also observed in infected cells pre-treated with the NF-κB inhibitor BAY 11–7085 (40 μM) (Figure 2C). Thus, NF-κB activity appeared to be crucial for viral APOBEC3G induction.

To independently analyse whether NF-κB might play a role in APOBEC3G induction, we stimulated cells with TNFα (20 ng/ml), a very strong activator of NF-κB [27]. However, TNFα stimulation did not result in enhanced APOBEC3G gene transcription (data not shown), indicating that NF-κB activity alone is not sufficient to induce human APOBEC3G gene transcription.

Influenza virus infection results in type I IFN production (Figure 2B) and subsequent expression of IFN-responsive genes [28-30]. So far, it was not clear from the literature whether human APOBEC3 genes are induced by type I IFNs. While IFN-dependency was reported for the hepatoma cell lines HepG2 and Huh7 [18,31] and for macrophages [32], human APOBEC3 proteins are not inducible in H9 cells by type I and type II IFN [23]. To specifically address this issue for the lung epithelial cell line used in our study, A549 cells were incubated for different time periods with recombinant IFNβ (100 U/ml) (PBL), and the levels of human APOBEC3G and human APOBEC3F mRNAs were determined by qRT-PCR (Figure 2D).

IFNβ stimulation led to a nearly 20-fold induction of the human APOBEC3G mRNA (Figure 2D), which could also be observed at the protein level (Figure 2E); by contrast, the human APOBEC3F mRNA was not affected at all (Figure 2D). Strikingly, this pattern of human APOBEC3G versus human APOBEC3F expression exactly matched the results obtained upon virus infection (Figure 1B). This suggests that IFNβ, expressed upon virus infection in an NF-κB dependent manner, may be an indirect trigger of human APOBEC3G expression, leaving still open the question about the initial viral inducer.

**Figure 3:**
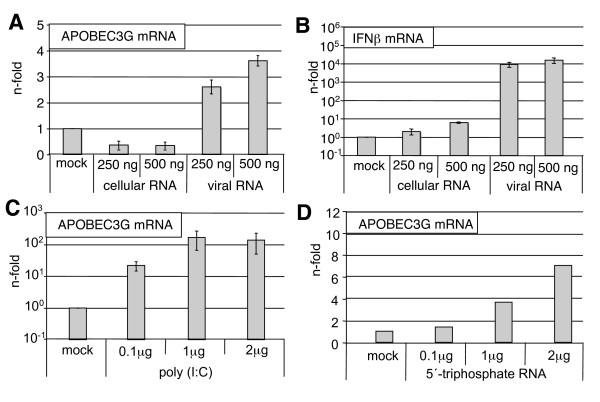
**Induction of human APOBEC3G mRNA by different RNA species**. (A and B) A549 cells (1.5 × 10^6^/6 cm dish) were transfected with indicated amounts of total RNA from virally infected ("viral RNA") or uninfected ("cellular RNA") A549 cells. RNA from uninfected or virally infected cells was generated by isolation of total RNA from cells either infected with PR8 (MOI = 5) or from uninfected cells using the Qiagen RNeasy kit according to the manufacturer's instructions. For stimulation, these RNAs were transfected for 10 hours using Lipofectamine 2000 (L2000) (Invitrogen) according to the manufacturer's protocol. (C) A549 cells were transfected with either different amounts of poly (I:C) (Amersham Biosciences) or with 5'-triphosphate RNA (D). 5'-triphosphate RNA was generated by reverse transcription of a short PCR product using the MEGAshortscript kit (Ambion) according to the manufacturer's instructions. mRNA levels of IFNβ (B) or human APOBEC3G (A, C and D) were determined by qRT-PCR as described. Levels of gene induction were calculated as n-fold of the background of untreated cells, which was arbitrarily set as 1.

IFNβ transcription in infected cells is known to be mainly induced by single-stranded or partially double-stranded RNA. Such RNA species accumulate during infection within the host cell and serve as a pathogen pattern sensed by cells [33,34]. To examine whether different RNA species serve as inducer of APOBEC3G gene expression, total RNAs isolated from influenza virus infected ("viral RNA") or uninfected cells ("cellular RNA"), or the dsRNA analogue poly (I:C), or short ssRNA bearing a 5'-triphosphate were used as stimuli to elicit a gene response. These RNAs were transfected into A549 cells, and mRNA levels of human APOBEC3G and IFNβ were determined (Figure 3A–D). While transfection of RNA from uninfected cells led to no significant gene induction, RNA from virally infected cells resulted in upregulation of both, human APOBEC3G and IFNβ transcription (Figure 3A and 3B). Stimulation using either poly (I:C) or 5'-triphosphate RNA led to even a stronger induction of APOBEC3G (Figure 3C and 3D). In summary, our findings indicate that human APOBEC3G is induced upon viral infection as a part of the antiviral response mediated by type I IFN. This response is triggered by the recognition of different RNA species by distinct receptors such as TLR3, RIG-I and/or MDA-5. Interestingly, we did not observe any human APOBEC3F induction, neither upon viral infection nor with IFNβ stimulation (Figure 1B and 2D), albeit both promoters carry ISRE elements [35]. Thus, we hypothesized that human APOBEC3G may be selectively induced and may confer a specific antiviral activity in influenza virus infected cells.

**Figure 4:**
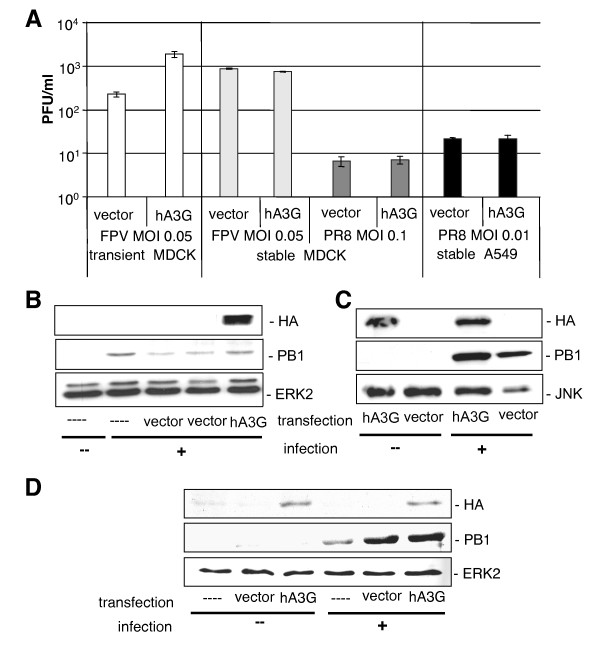
**Influence of the human APOBEC3G protein on viral replication**. (A) Transiently (white bars) or stably human APOBEC3G transfected (light and dark grey bars) MDCK cells were infected with avian influenza virus A/FPV/Bratislava/79 (H7N7) (FPV) (MOI = 0.05) for 9 hours or A/Puerto Rico/8/34 H1N1 (PR8) (MOI = 0.1) virus for 16 hours. Both virus strains were originally taken from the collection of the Institute of Virology, University of Giessen, Germany. Stably transfected A549 cells (black bars) were infected with PR8 for 16 hours (MOI = 0.01). Supernatants were taken, and virus titres were determined by means of plaque assay. (B and C) MDCK cells or (D) A549 cells were transfected with 3 μg DNA/6 well dish pcDNA-APOBEC3G or pcDNA3.1 empty vector using L2000 (Invitrogen) according to manufacturer's instructions. To generate a bulk amount of stable expressing cells, MDCK cells (C) or A549 cells (D) were treated with G418 300 μg/ml for selection of APOBEC3G expression for four weeks. Thereafter, cells were infected with FPV at MOI = 0.05 (B) or at MOI = 0.1 for 9 hours (C) or with PR8 at MOI = 0.001 for 16 hors (D). Expression of HA-tagged human-APOBEC3G was detected by anti-HA 3F10 (Roche) antibody. To control equivalent protein loading, ERK2 or JNK were detected by anti-ERK2 (Santa Cruz) or anti-JNK antibody (Santa Cruz). Viral replication was monitored by detection of the viral polymerase protein using an anti-PB1 antibody (Santa Cruz).

To test this assumption we first transiently over expressed HA-tagged human APOBEC3G (Figure 4B) and assessed the efficiency of viral propagation in these cells. Surprisingly, in the presence of human APOBEC3G, progeny virus titres were slightly elevated compared to the vector control (Figure 4A, white bars). This correlated with a slightly higher expression level of the viral polymerase subunit PB1 (Figure 4B). To circumvent potential transient transfection artefacts and to enhance the number of transgene-expressing cells, we generated cell lines, stably expressing human APOBEC3G (Figure 4C and 4D). After selection of stably APOBEC3G expressing cells by antibiotic treatment, the cells were infected with different influenza A virus strains at various multiplicities of infection (MOI) (Figure 4A, grey and black bars). In contrast to the transient situation, viral propagation was not affected in these stably transfected cells, although the transgene was expressed well in MDCK cells (Figure 4C) as well as in A549 cells (Figure 4D). Thus, although influenza A virus induces human APOBEC3G transcription in an NF-κB and IFNβ dependent manner, the forced expression of human APOBEC3G did not result in any antiviral effect on this virus. This is different from the situation with HIV-1. APOBEC3G shows antiviral activity against HIV-1 and other retroviruses [8,2]. However, HIV-1 does not induce APOBEC3G transcription in cell culture [2,3]. In support of a specific rather than broad antiviral activity of APOBEC3G, Kremer et al. [36] had reported that overexpressed human APOBEC3G also has no antiviral effect against vaccinia virus (VACV). In summary, we conclude that human APOBEC3G is induced by influenza A viral RNA, via an NF-κB dependent mechanism as part of the antiviral IFN response program but does not exhibit an antiviral effect against influenza A virus.

## Competing interests

The authors declare that they have no competing interests.

## Authors' contributions

EKP, MS, CE and HH have performed experimental work, contributed data and gave conceptual input in the study design. EF and CM have provided important material and have been involved in drafting the manuscript and revising it critically for important intellectual content. SL has designed and has guided the study, interpreted the data and wrote the manuscript.
